# Near-Complete Genome Sequences of Measles Virus Strains from 10 Years of Uganda Country-wide Surveillance

**DOI:** 10.1128/mra.00606-22

**Published:** 2022-07-25

**Authors:** Prossy Namuwulya, Henry Bukenya, Phionah Tushabe, Robert Tweyongyere, Josephine Bwogi, Matthew Cotten, My V. T. Phan

**Affiliations:** a Uganda Virus Research Institute, Entebbe, Uganda; b Department of Veterinary Pharmacy Clinical and Comparative Medicine, Makerere University, Kampala, Uganda; c U.K. Medical Research Council-Uganda Virus Research Institute and London School of Hygiene and Tropical Medicine Uganda Research Unit, Entebbe, Uganda; d U.K. Medical Research Council-University of Glasgow Centre for Virus Research, Glasgow, Scotland, United Kingdom; KU Leuven

## Abstract

Measles remains a global health challenge despite the availability of a safe and effective vaccine. Sporadic outbreaks of measles virus infections continue in Uganda. We report eight near-complete genome sequences of measles virus strains from Uganda cases from 2011 to 2020, providing useful data for assessing vaccine escape and local/regional transmission.

## ANNOUNCEMENT

Measles virus (MV) is classified in the *Measles morbillivirus* species (genus *Morbillivirus*, family *Paramyxoviridae*) and is encoded by a negative-sense single-stranded RNA linear genome of about 16,000 nucleotides (nt). Measles is a very transmissible viral infection and, despite an effective vaccine, MV continues to infect and sicken individuals throughout the world. Disruptions of routine vaccination by the coronavirus disease 2019 (COVID-19) epidemic are resulting in further increases in global measles cases (1). The currently used MV vaccines are based on a genotype A MV isolate from 1954 (2). Continued MV evolution may erode the efficacy of the vaccines currently in use, requiring adjustments in vaccines or vaccination schedules for protection against currently circulating MV strains. Accordingly, full genome sequences of MV strains isolated from contemporary measles cases from all parts of the world are essential for keeping ahead of this pathogen. We have developed a simple amplicon-based sequencing method suitable for the MinION sequencing platform to generate genome sequences of MV strains, which is especially functional in resource-poor settings. Here, we report eight near-complete genome sequences from Uganda cases from 2011 to 2020, addressing the lack of MV full genomes from Uganda and from East Africa.

MV IgM enzyme-linked immunosorbent assay (ELISA)-positive cases (3) were identified by Expanded Programme on Immunization (EPI) MV surveillance activity in Uganda (4), and throat swab samples from IgM-positive cases were cultured on Vero/hSLAM cells (5) for 1 or 2 passages of 5 days. The MV-positive cultures were sequenced using MV-specific genotype B3 amplicon primers that amplify the full-length MV genome (primer sequences are available at https://github.com/mlcotten13/Measles_primers). Briefly, total viral RNA was extracted from cell culture material using QIAamp viral RNA minikits following the manufacturer’s instructions. MV RNA was reverse transcribed using amplicon-specific primers and SuperScript III reverse transcriptase (Invitrogen), followed by PCR amplification using Phusion high-fidelity DNA polymerase (Thermo Fisher Scientific). PCR amplicon products were subjected to MinION sequencing library preparation using the SQK-LSK109 barcoding kit (Oxford Nanopore Technologies) according to the manufacturer’s instructions, followed by sequencing on MinION SpotON R9.4.1 flow cells. The resulting fast5 files were base called and demultiplexed using Oxford Nanopore Technologies Guppy software v.5.0.11+2b6dbff (6). Demultiplexed reads for each sample were used for reference-based assembly using minimap2.1 (7) with MVs/California.USA/34.19/[B3] (GenBank accession number MT789820) as the reference sequence. Consensus genome sequences and final sequence checks were determined using Geneious Prime v.2022.1.1 (8). All tools were run with default parameters unless otherwise specified. Differences or ambiguities in the genome sequences were resolved manually by counting coverage with quality-controlled read data. Details for the samples are found in Table 1, including case dates and locations, sequencing metrics, and GenBank and SRA accession numbers for the sequence data.

**TABLE 1:** Sampling details, assembly statistics, and accession numbers for the strains in this study

Sample identifier	Strain	BioProject accession no.	SRA accession no.	GenBank accession no.	Location	Collection date (yr-mo-day)	No. of raw reads^*a*^	No. of MV-specific reads	Genome length (nt)	GC content (%)	No. of nucleotide differences from reference^*b*^	Identity to reference (%)^*c*^	No. of nucleotide differences from earliest Uganda strain^*d*^	Identity to earliest Uganda strain (%)^*e*^
GEMVI_11_2	MVi/Kyenjojo.UGA/24.13 [B3]	PRJNA843031	SRX15483626	ON642794	Kyenjojo	2013-6-11	183,718	170,266	15,657	47.1	349	97.76	20	99.88
GEMVI_11_3	MVi/Otuke.UGA/13.19 [B3]	PRJNA843031	SRX15483627	ON642795	Otuke	2019-3-28	283,310	256,334	15,700	47.1	174	98.88	354	97.72
GEMVI_11_4	MVi/Kole.UGA/05.20 [B3]	PRJNA843031	SRX15483628	ON642796	Kole	2020-1-30	426,125	393,265	15,709	46.5	193	98.76	373	97.60
GEMVI_12_9	MVi/Jinja.UGA/50.11 [B3]	PRJNA843031	SRX15483629	ON642797	Jinja	2011-12-7	120,874	106,074	15,624	47.6	338	98.82	0	100
GEMVI_12_12	MVi/Kabarole.UGA/13.13 [B3]	PRJNA843031	SRX15483630	ON642798	Kabarole	2013-3-26	298,611	273,575	15,645	47.6	344	97.79	14	99.92
GEMVI_12_18	MVi/Kampala.UGA/32.17 [B3]	PRJNA843031	SRX15483631	ON642799	Kampala	2017-8-10	167,430	165,049	15,710	47.4	164	98.95	341	97.81
GEMVI_12_20	MVi/Bushenyi.UGA/01.12 [B3]	PRJNA843031	SRX15483632	ON642800	Bushenyi	2012-1-2	144,609	129,043	15,543	47.1	348	97.76	30	99.81
GEMVI_12_23	MVi/Hoima.UGA/01.13 [B3]	PRJNA843031	SRX15483633	ON642801	Hoima	2013-1-4	1,588,359	1,387,323	15,678	47.4	350	97.75	20	99.87

a Read lengths of 2,000 to 2,500 nt.

b Number of nucleotide differences from the genome of MVs/California.USA/34.19/[B3] (GenBank accession number MT789820).

c Percentage of nucleotide differences from the genome of MVs/California.USA/34.19/[B3] (GenBank accession number MT789820).

d Number of nucleotide differences from the earliest Uganda MV strain (sample identifier GEMVI_12_9) near-complete genome (GenBank accession number ON642797).

e Percentage of nucleotide differences from the earliest Uganda MV strain (sample identifier GEMVI_12_9) near-complete genome (GenBank accession number ON642797).

The eight near-complete MV genome sequences reported here were obtained from Uganda measles cases identified in 2011 to 2020 from locations across the country. Despite small numbers, these eight sequences help address the lack of MV genome sequences from Uganda and from East Africa, where outbreaks of measles occur regularly. The sequences were all classified as genotype B3, the genotype most frequently reported from Africa (9, 10). The MV sequencing primers and method for the MinION platform reported here provide a useful tool for monitoring MV evolution and transmission.

The study was approved by the Uganda Ministry of Health (reference number 105/197/01), the Uganda Virus Research Institute Research and Ethics Committee (reference number GC/127/19/12/740), and the Uganda National Council for Science and Technology (reference number HS2741).

### Data availability.

The genome sequences described here have been deposited in GenBank with the accession numbers ON642794 to ON642801. The unassembled read data are available in the SRA with the accession numbers SRX15483626 to SRX15483633 under BioProject accession number PRJNA843031 (Table 1). Primer sequences are available at https://github.com/mlcotten13/Measles_primers.
